# Metformin Induces Apoptosis and Downregulates Pyruvate Kinase M2 in Breast Cancer Cells Only When Grown in Nutrient-Poor Conditions

**DOI:** 10.1371/journal.pone.0136250

**Published:** 2015-08-20

**Authors:** Alessandra Silvestri, Francesco Palumbo, Ignazio Rasi, Daniela Posca, Theodora Pavlidou, Serena Paoluzi, Luisa Castagnoli, Giovanni Cesareni

**Affiliations:** 1 Laboratory of Molecular Genetics, Department of Biology, Tor Vergata University, Rome, Italy; 2 IRCCS, Fondazione Santa Lucia, Rome, Italy; National Cancer Center, JAPAN

## Abstract

**Introduction:**

Metformin is proposed as adjuvant therapy in cancer treatment because of its ability to limit cancer incidence by negatively modulating the PI3K/AKT/mTOR pathway. *In vitro*, in addition to inhibiting cancer cell proliferation, metformin can also induce apoptosis. The molecular mechanism underlying this second effect is still poorly characterized and published data are often contrasting. We investigated how nutrient availability can modulate metformin-induced apoptosis in three breast cancer cell lines.

**Material and Methods:**

MCF7, SKBR3 and MDA-MB-231 cells were plated in MEM medium supplemented with increasing glucose concentrations or in DMEM medium and treated with 10 mM metformin. Cell viability was monitored by Trypan Blue assay and treatment effects on Akt/mTOR pathway and on apoptosis were analysed by Western Blot. Moreover, we determined the level of expression of pyruvate kinase M2 (PKM2), a well-known glycolytic enzyme expressed in cancer cells.

**Results:**

Our results showed that metformin can induce apoptosis in breast cancer cells when cultured at physiological glucose concentrations and that the pro-apoptotic effect was completely abolished when cells were grown in high glucose/high amino acid medium. Induction of apoptosis was found to be dependent on AMPK activation but, at least partially, independent of TORC1 inactivation. Finally, we showed that, in nutrient-poor conditions, metformin was able to modulate the intracellular glycolytic equilibrium by downregulating PKM2 expression and that this mechanism was mediated by AMPK activation.

**Conclusion:**

We demonstrated that metformin induces breast cancer cell apoptosis and PKM2 downregulation only in nutrient-poor conditions. Not only glucose levels but also amino acid concentration can influence the observed metformin inhibitory effect on the mTOR pathway as well as its pro-apoptotic effect. These data demonstrate that the reduction of nutrient supply in tumors can increase metformin efficacy and that modulation of PKM2 expression/activity could be a promising strategy to boost metformin anti-cancer effect.

## Introduction

Metformin (1,1-dimethylbiguanide hydrochloride) is the first-choice oral therapy in patients with type-II diabetes [1,2]. Aside from the main impact of metformin on glucose metabolism in the liver, other effects of the drug have been reported, such as the diabetes prevention in pre-diabetic subjects [3], the ability to reduce the vascular complications associated with diabetes [4] and the ability to reactivate the ovary function in the polycystic ovary syndrome [5]. Moreover, epidemiological, clinical and preclinical evidences indicated an important role of metformin in the prevention of cancer [6]. Evans et al. reported a correlation between a metformin regimen and a reduced cancer incidence [7]. These observations have been validated by a recent study showing a 31% reduction in cancer incidence and mortality when metformin is administered in the treatment of diabetes [8]. Moreover, because of decreasing insulin levels, metformin-treated patients present a reduced risk of developing obesity related tumors, such as breast and colorectal cancers [9].

Even if the beneficial effect of metformin on tumor development has received considerable support, the molecular mechanism underlying its anti-cancer activity is poorly understood. The drug directly targets the mitochondrial respiratory complex 1 thereby affecting the AMP/ATP balance and activating the AMP kinase (AMPK) [10,11]. Active AMPK in turn phosphorylates a series of effectors inducing the inhibition of pathways requiring ATP consumption such as fatty acids and protein synthesis [12]. One of these effectors is TSC2 that, upon phosphorylation, causes a reduction of mTOR signalling [13], which controls protein synthesis and regulates the phosphorylation of different proteins such as p70S6K, 4E-BP1 and the protein synthesis initiation factor, eIF4G. By inhibiting this signalling pathway, metformin induces a general reduction in protein synthesis therefore affecting cell growth and proliferation.

Recently, it was suggested that metformin not only inhibits proliferation but also it induces apoptosis. In 2009 it was reported that metformin reduces cell growth and proliferation by inducing cell cycle arrest in MCF7 and SKBR3 cells [14] while it increases apoptosis in MDA-MB-231 cells [15]. Controversially, in 2010 Zakikhani et al demonstrated that after 72 hours treatment with metformin MCF7 breast cancer cells were positive to Annexin V staining and PARP inactivation, two classical markers of apoptosis [16]. Moreover, in 2011 Zhuang and Miskimins demonstrated that metformin was able to induce up to 50% cell death in MCF7 cells while, in MDA-MB-231 cells, the apoptotic level was lower that 3% [17].

Finally, recent data highlighted a correlation between glucose availability and metformin pro-apoptotic effect. While in cells grown in high-glucose medium the drug induced only cell cycle arrest, in cells maintained in a glucose-free medium it was possible to observe a marked apoptotic effect [18]. These observations have been confirmed by two additional reports in which the authors demonstrated that glucose availability strongly influences metformin ability to induce cell cycle arrest [19] and to decrease cell proliferation [20].

Considering the available literature that reports dissimilar results even in the same cell line [14–17], the aim of our work was to clarify how experimental conditions could influence the cellular response to metformin treatment. To this end, we tested whether and how nutrient availability could modulate metformin-induced inhibition of the mTOR pathway and promote apoptosis.

## Materials and Methods

### Cell lines and culture conditions

Human breast cancer cell lines MCF-7, SK-BR3 and MDA-MB-231 were obtained from American Type Culture Collection (ATCC, Rockville, MD). MCF-7 were maintained in Minimum Essential Medium (MEM) (Gibco, Life Technologies), supplemented with sodium pyruvate 1mM, Penicillin/Streptomycin 100 U/ml-100 μg/ml, insulin 0,01 mg/ml and 10% FBS. SK-BR3 and MDA-MB-231 cells were grown in RPMA medium (Gibco, Life Technologies) supplemented with Penicillin/Streptomycin 100 U/ml-100 μg/ml and 10% FBS. All cells were maintained in a 37° humidified atmosphere containing 95% air and 5% CO_2_.

### Cell treatment

For treatment cells were plated in MEM medium supplemented with increasing glucose concentrations or in DMEM medium. 24 hours after plating, cells were treated for different time lengths with the following drugs: metformin (Sigma-Aldrich) 10 mM, rapamycin (Sigma-Aldrich) 100 nM and AICAR (Sigma-Aldrich) 3 mM. For the 48 hours treatment, 10 mM metformin was added every 24 hours. The control samples were treated with an equal quantity of PBS.

### Trypan Blue exclusion assay

Cells were plated in 6-well plates and treated as indicated in each figure. After treatment cells were harvested by trypsinization and stained using a 0.2% Trypan Blue solution. Trypan Blue positive and negative cells were counted using a hemocytometer and the number of dead cells was calculated as percentage of dead cells on the total number of cells. All the experiments were done in triplicate and the results are indicated as a mean +/- standard deviation of three independent replicates.

### Western Blotting analysis

Cells were lysed using Cell Signaling Lysis Buffer (Millipore) plus protease and phosphatase inhibitor cocktails (Sigma-Aldrich) and total protein content was measured using Bradford assay. The antibodies used for the detection of the total or phosphorylated forms of the protein of interest were the following: RPS6K, phospho-RPS6K (Ser240/244), mTOR, phospho-mTOR (Ser2448), Caspase 7, Cleaved Caspase-7 (Asp198), PARP, Cleaved PARP (Asp214) and PKM2. All the antibodies were purchased from Cell Signaling Technology. Anti-GAPDH (Millipore) was used as loading control.

### Apoptosis determination by Annexin V/Propidium Iodide (PI) assay

For apoptosis determination, cells were analyzed by flow cytometry after staining with Annexin V/PI following manufactures instructions (Cell Signaling Technology). In brief, after treatment, adherent and floating cells were collected, washed with ice-cold PBS and resuspended in Annexin V binding buffer. 1 μL of Annexin V-FITC Conjugate and 12.5 μL of PI were added to each sample and samples were incubated 10 minutes on ice protected from light. Stained cells were diluted in ice-cold buffer and directly analyzed by FACSCalibur cytofluorimeter.

This double staining allows highlighting four dinstinct cell populations: alive cells (annexin V negative, PI negative), early apoptotic cells (annexin V positive, PI negative), late apoptotic cells (annexin V positive, PI positive) and necrotic cells (annexin V negative, PI positive). Cell percentage for each population was determined using FlowJo sofware (FlowJo, LLC, USA). Annexin V/PI analysis was done on two independent biological replicates and reported results are indicated as a mean +/- standard deviation.

### Real-time PCR

Total RNA was extracted from cells after treatment using RNeasy kit (Qiagen). RNA was eluted in 35 μL RNase-free water. Total RNA was quantified by Nanodrop and 0,2–2 μg were reverse transcribed with High Capacity cDNA Reverse Transcription Kits (AppliedBiosystem); reaction was performed in a final volume of 20 μL. 10–30 ng cDNA was amplified using Power SYBR Green PCR Master Mix in StepOne System (AppliedBiosystem) in a final volume of 20μL. To analyze PKM2 following primers were used: Forward TGACGAGAACATCCTGTGGC, Reverse GGAAGTCGGCACCTTTCTGC. Beta-actin was used as endogenous control for sample normalization using the following primers: Forward ACCACCATGTACCCTGGCATT, Reverse CCACACGGAGTACTTGCGCTCA. Each experiment was done in in triplicate and the results are indicated as a mean +/- standard deviation of three independent biological replicates.

### Statistical analysis

Each experiment was analysed in three biological replicates. For the molecular analysis images are representative of the three independent experiments. For the analysis of the percentage of dead cells, graphs report the mean of the experiments +/- standard deviation. Statistical analysis was performed using a two sided student’s t-test. Data were considered statistically different when p ≤ 0.05 (* = p ≤ 0.05, ** = p ≤ 0.01, *** = p ≤ 0.001). Statistics was performed using Graph Pad Prism Version 6.

## Results

### Metformin effects on the mTOR pathway and apoptosis are affected by plating density

**Fig 1:**
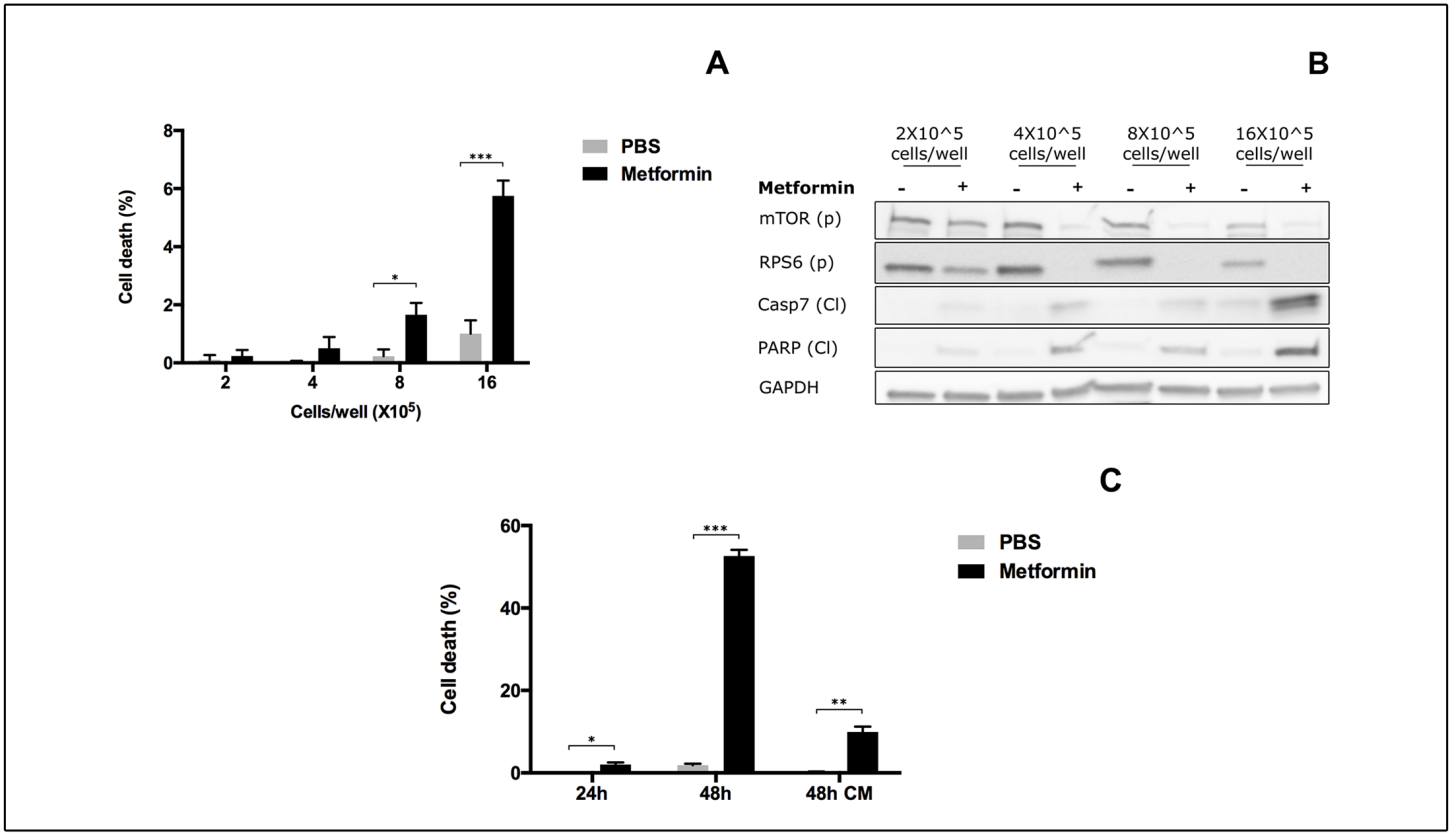
Cell density and nutrient availability influence metformin effect on mTOR pathway and apoptosis. MCF7 cells were plated in MEM medium at different densities from 2X10^5^ to 16X10^5^ cell/well in 6-well plates. Cells were then treated with 10mM metformin or with PBS as control for 24 hours. **A**) After treatment cells were collected and counted with Trypan Blue dye to discriminate between dead and alive cells. Results are reported as percentage of dead cells on the number of total cells. The bars represent the mean of three independent experiments. **B**) Cells were collected after 24 hours treatment and protein extracts were subjected to Western Blot analysis to determine the level of phosphorylation of mTOR (p) Ser2448 and RPS6 (p) Ser240/244. We also monitored the levels of cleaved Caspase7 (Cl) and PARP (Cl). GAPDH was used as loading control. The blot is representative of three independent biological replicates. **C**) MCF7 cells were plated in MEM medium at a density of 8X10^5^ cells/well and treated with 10 mM metformin or with PBS for 24h, 48h or 48h changing the medium after the first 24h treatment (48h CM). Cells were collected after treatment and counted with Trypan Blue dye to discriminate dead from alive cells. Results are reported as percentage of dead cells over the total number of cells. The bars represent the mean of three independent experiments.

We first noticed that cell density influences the response of MCF7 breast cancer cells to metformin treatment. Cells were plated at densities ranging from 2X10^5^ to 16X10^5^ cells in 6 well plates in MEM medium and treated with 10 mM metformin for 24 hours. Trypan Blue staining revealed a correlation between the percentage of cell death and initial plating density (Fig 1A). To obtain hints about the underlying molecular mechanism we investigated whether, the inactivation of the mTOR pathway, an established target of metformin treatment, was also influenced by plating density. Cells treated with metformin were lysed and protein extracts were analysed by Western Blot to determine the levels of the activated form of phospho-mTOR (mTOR (p)) and of its substrate, phospho-RPS6 (RPS6 (p)). Both readouts indicated that the inhibitory effect of metformin on the mTOR pathway is dependent on cell density (Fig 1B). As control for the decrease in mTOR and RPS6 phosphorylation, total protein expression was analysed and results are shown in S1 Fig. Interestingly, while no variation in mTOR expression after 24 hours of treatment was present, RPS6 expression was increased in cells plated at high densities. This result indicates that metformin inhibition of RPS6 activity is even stronger in this condition since it is maintained even after an increase in total protein expression.

We next focused on the ability of metformin to induce apoptosis by monitoring the levels of cleaved-PARP (PARP (Cl)) and cleaved-Caspase7 (Casp7 (Cl)). As shown in Fig 1B, cells plated at low density (2X10^5^ cells/well), if treated with metformin, did not show detectable amounts of cleaved caspase-7 or PARP indicating that in this experimental condition metformin was not able to induce apoptosis in our cellular model (Fig 1B). Conversely, when cells were plated at higher densities it was possible to detect the apoptotic forms of the two proteins, confirming that high cell density increases metformin cytotoxicity. In all conditions considered, the total forms of Caspase 7 and PARP were not altered after 24 hours treatment (S1 Fig). Since in the highest plating density condition (16X10^5^ cells/well), cells were showing signs of “sufferance” irrespective of drug treatment, as indicated by the increasing percentage of dead cells and the appearance of apoptotic molecular readouts in the control samples, we selected the 8X10^5^ cells/well condition for the following experiments. In this condition only 2% of the cells stain with Trypan Blue after 24 hours of metformin treatment. This percentage rises to approximately 50% after 48 hours. However, this effect is limited if the growth medium is replenished with fresh medium after the first 24 hours incubation suggesting that some essential component of the MEM medium is used up in the initial 24 hours (Fig 1C).

### Glucose availability influences metformin effect on apoptosis without affecting its ability to downregulate the mTOR pathway

**Fig 2:**
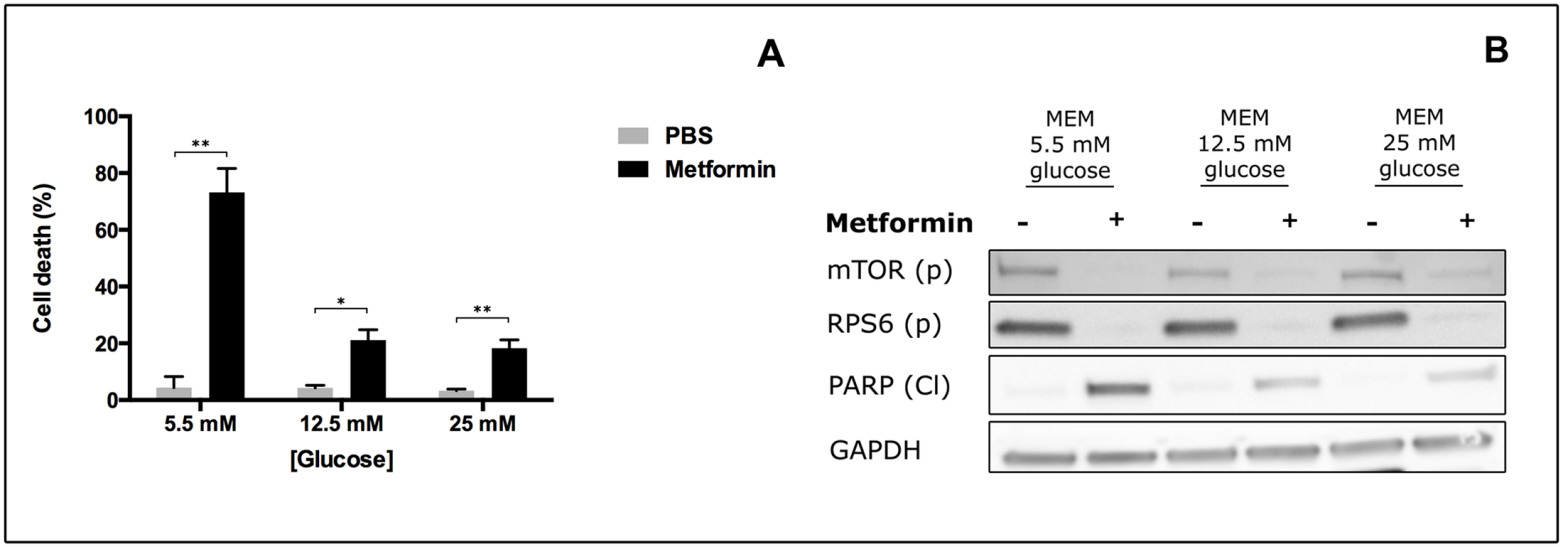
Glucose availability influences metformin effect on apoptosis without affecting mTOR pathway downregulation. MCF7 cells were plated at 8X10^5^ cells/well in 6-well plates in MEM medium with increasing amounts of glucose from 5.5 mM to 25 mM and treated with 10 mM metformin for 48 hours. A) After treatment cells were collected, stained with Trypan Blue to discriminate between dead and alive cells and counted. Results are reported as percentage of dead cells on the number of total cells. Data reported is a mean of three independent experiments. B) After treatment cells were lysed and protein extracts were analysed by Western Blot with antibodies directed against phosphorylated mTOR (p) Ser2448, RPS6 (p) Ser240/244 and cleaved PARP (Cl). GAPDH was used as loading control.

We next asked whether glucose depletion is the cause of the toxic effect of metformin. Cells were plated at increasing glucose concentration, from 5.5 mM to 25 mM, and treated with 10 mM metformin for 48 hours. The percentage of dead cells was then determined by Trypan Blue staining and the effect of the treatment on the mTOR pathway as well as on apoptosis was monitored by Western Blot. Cell death was significantly mitigated when the glucose concentration was 12.5 mM or higher (Fig 2A) and PARP cleavage was reduced at higher glucose concentrations (Fig 2B). However, the metformin induced decrease in activation of the mTOR pathway was still observed at high glucose concentration. This suggests that glucose availability has a role in metformin-induced apoptosis but it does not affect the downregulation of the mTOR pathway.

For control purposes, total protein levels are reported in S2 Fig. The increase in RPS6 total protein expression observed after 24 hours treatment (S1 Fig) was maintained after prolonged treatment. Interestingly, in low nutrient medium this effect was less pronounced suggesting that even if cells upregulates RPS6 protein expression to contrast metformin inhibitory effect on mTOR pathway, the constant inhibition of its activity cause protein degradation. After 48 hours treatment, in fact, not only RPS6 but also mTOR started to be downregulated in low nutrient medium indicating that the entire pathway is affected by prolonged treatment.

### DMEM, a nutrient-rich medium, abolishes cytotoxicity induced by metformin and reduces metformin effect on the mTOR pathway

**Fig 3:**
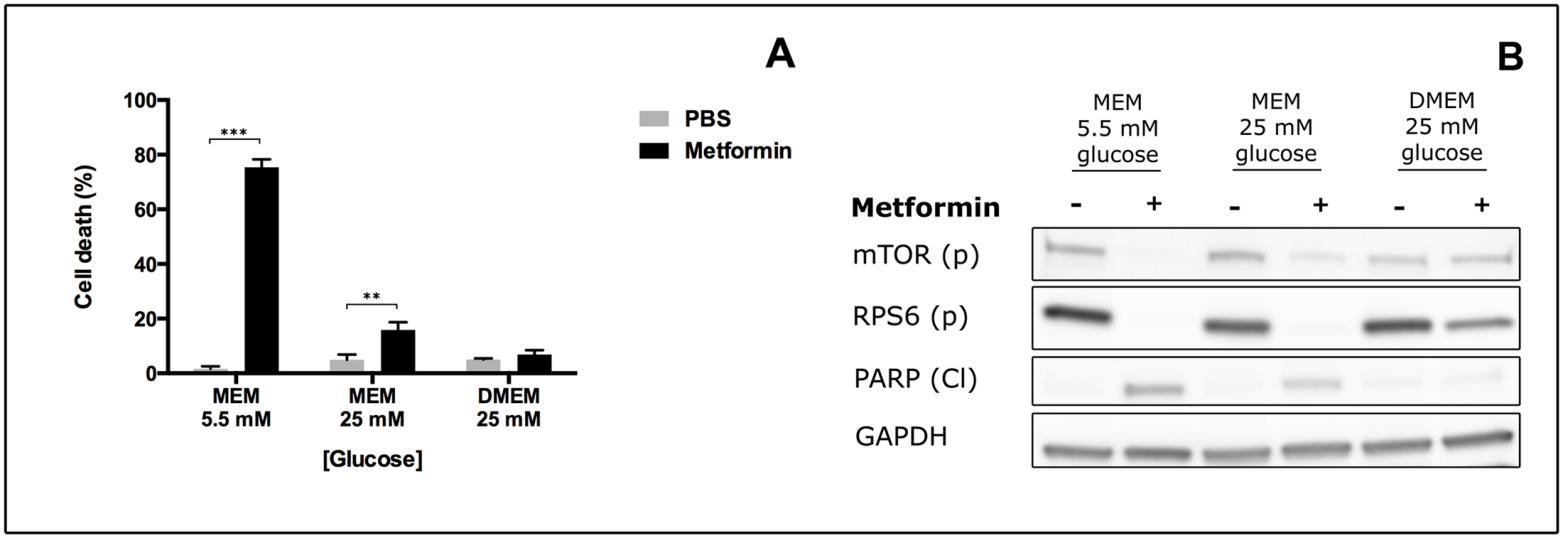
Metformin downregulation of mTOR pathway and induction of apoptosis are negatively modulated in nutrient-rich medium. MCF7 cells were plated at 8X10^5^ cells/well in 6-well plates in MEM medium with 5.5 mM or 25 mM glucose or DMEM and treated with 10 mM metformin for 48 hours. A) After treatment cells were trypsinized and counted after staining with the Trypan Blue dye to discriminate dead from alive cells. Results are reported as percentage of dead cells on the number of total cells. Data reported is the mean of three independent experiments. B) After treatment cells were lysed and protein extracts were analysed by Western Blot with antibodies directed against mTOR (p) Ser2448, RPS6 (p) Ser240/244 and PARP (Cl). GAPDH was used as loading control.

In laboratory settings, cell lines are often grown in nutrient-rich media in which, not only glucose, but also amino acid concentrations are high if compared to physiological conditions. To determine whether the use of nutrient-rich growth media influences metformin effect on MCF7 cells, we compared the effect of the treatment in cells grown in standard MEM medium (MEM 5.5 mM glucose), in MEM medium supplemented with 25 mM glucose or in the nutrient-rich DMEM medium (25 mM glucose + amino acid enrichment). As shown in Fig 3A, when cells were grown and treated in DMEM, the percentage of cells positive to Trypan Blue staining was significantly decreased if compared to MEM medium. Moreover, the cytotoxic effect of metformin on cells grown in DMEM was lower then that on cells grown in MEM medium supplemented with 25 mM glucose, indicating that the presence of additional nutrients, other then glucose, can rescue cells from metformin-induced apoptosis (Fig 3A). Moreover, cells grown in DMEM are less sensitive to metformin effects on both the mTOR and the apoptotic pathways. As shown in Fig 3B, while the treatment with 10 mM metformin in MEM negatively affects the activity of mTOR, irrespective of glucose concentration, mTOR activity was not reduced in DMEM where the phosphorylation levels of mTOR and RPS6 were hardly affected. For control purposes, total protein levels are reported in S3 Fig. Interestingly, the increase in RPS6 total protein expression observed in MEM medium with increasing concentration of glucose was not present in DMEM indicating that the presence of other nutrients other then glucose allow cells to control mTOR pathway activity even in the presence of metformin.

In addition to the mTOR pathway, apoptosis was also differentially modulated by metformin in these diverse growth conditions. In fact, metformin ability to induce PARP inactivation was abolished in cells grown in DMEM indicating that in this experimental conditions MCF7 cells are not sensitive to metformin cytotoxicity (Fig 3B and S3 Fig).

To further confirm that metformin induced cell death was due to the ability of the drug to activate apoptotic mechanisms, cells were stained for Annexin V and Propidium Iodide. As shown in S4A Fig, upper panel and S4B Fig, right, after 24h treatment apoptosis was induced only in cells grown in low nutrient medium while there was no difference compared to control when cells were grown with higher concentrations of glucose or in high nutrient medium. The ability of metformin to induce apoptosis was even stronger after 48 hours treatment where almost 70% of cells grown in MEM medium stained positive for both markers Annexin V and PI. Indeed, cells in hyperglycemic medium and even more cells grown in DMEM, had a lower percentage of apoptotic cells after treatment (S4A Fig, lower panel and S4B Fig, left).

### Nutrients influence the ability of metformin to modulate the activity of the mTOR pathway and apoptosis in two additional models of breast cancer

**Fig 4:**
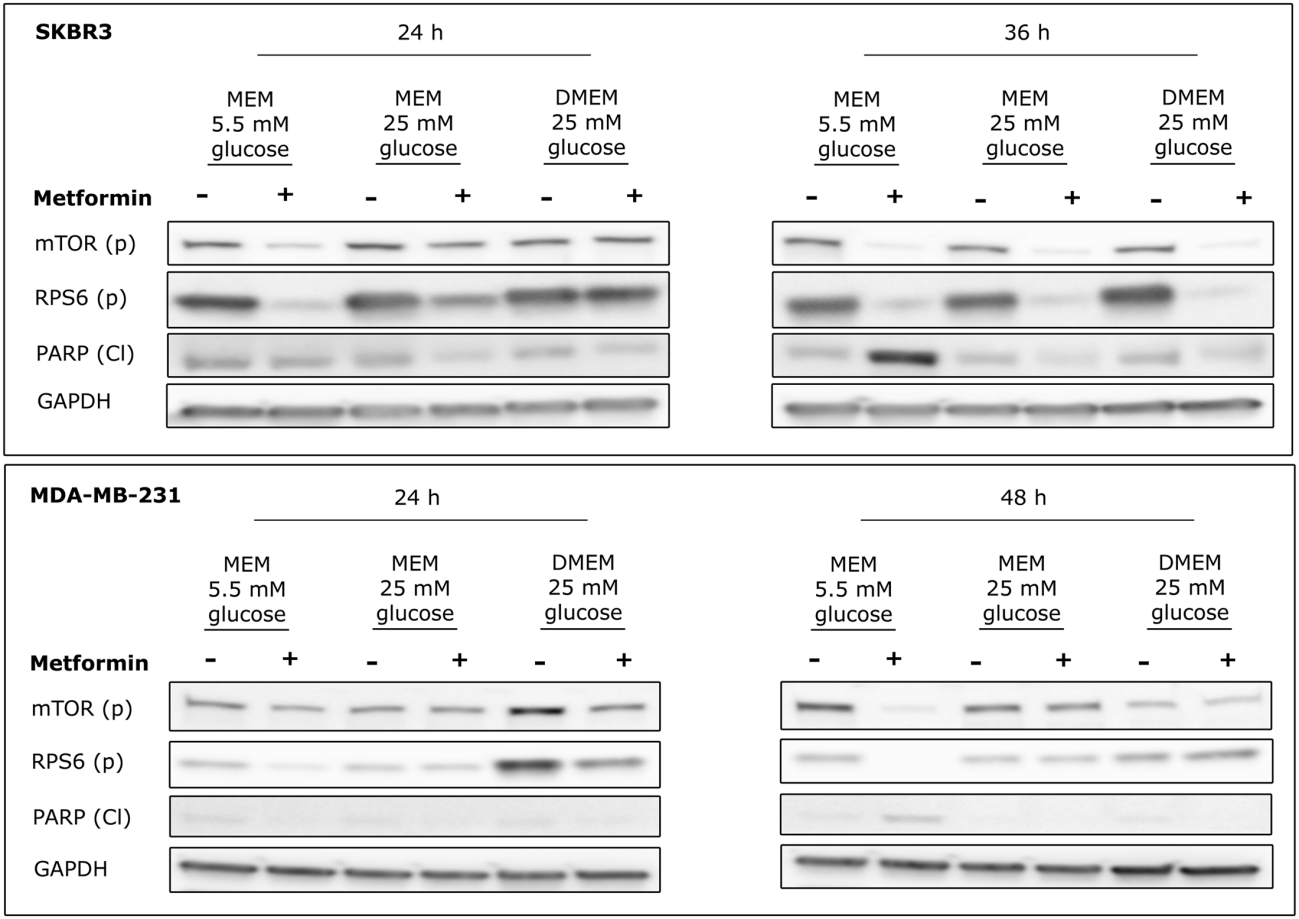
Effect of metformin on two additional models of breast cancer. SKBR3 and MDA-MB-231 cells were plated at 8X10^5^ cells/well in 6-well plates in different growth media (MEM 5.5 mM glucose, 25 mM glucose and DMEM) in 6-well plates treated with 10 mM metformin for 24h, and 36h or 48 hours, respectively. After treatment cells were lysed and protein extracts were analysed by Western Blot with anti-mTOR (p) Ser2448, RPS6 (p) Ser240/244 and PARP (Cl) antibodies. GAPDH was used as loading control.

To extend our observations, and to establish their generality, we investigated the effect of metformin on two additional cellular models of breast cancer, SKBR3 and MDA-MB-231. Cells were plated at 8X10^5^ cells/well in MEM medium at two glucose concentrations or in DMEM medium and treated with 10 mM metformin. The two cell lines displayed different sensitivities to metformin, as apoptosis was already evident in SKBR3 after 36 hours of treatment while the levels of cleaved PARP in MDA-MB-231 was barely detectable after 48 hours. Similarly to what was observed in MCF7, mTOR inactivation by metformin was dependent on nutrient availability. As shown in Fig 4, upper panel, 24 hours metformin treatment induced in SKBR3 an almost complete downregulation of mTOR (p) and RPS6 (p) in MEM medium, while this effect was only partial when glucose was incremented to 25 mM and completely abolished when cells were grown in DMEM. Moreover, after 24 hours treatment the apoptotic pathway was still inactive as shown by the low levels of PARP (Cl) that are not distinguishable from controls (Fig 4 and S5 Fig, upper panels).

In MDA-MB-231 we highlighted an even stronger dependence on experimental conditions (Fig 4 and S5 Fig, lower panels). After 48 hours treatment, metformin was able to completely inhibit the activity of the mTOR pathway only in MEM medium while it had no effect at high glucose concentrations or in DMEM. Moreover, the expression level of PARP (Cl) was still very low with a percentage of cell death close to zero (data not shown). These data confirmed that metformin cytotoxicity in breast cancer cells is modulated by nutrient availability and that more aggressive cell lines, such as the triple negative MDA-MB-231, are more resistant to metformin treatment.

The ability of metformin to activate cell apoptosis was confirmed in all cell models analysed (data not shown). SKBR3 were characterize by a strong positivity for Annexn V and PI already after 24 hours treatment confirming the higher sensibility of this cell line to metformin. On the contrary, MDA-MD-231 presented a lower percentage of apoptotic cells after 48 hours treatment compared to MCF7 even when cells were grown in nutrient poor medium, further supporting the aforesaid data indicating a higher resistance to the treatment for this cell type.

### Metformin treatment induces PKM2 downregulation in MCF7 and SKBR3 at limiting glucose concentrations

**Fig 5:**
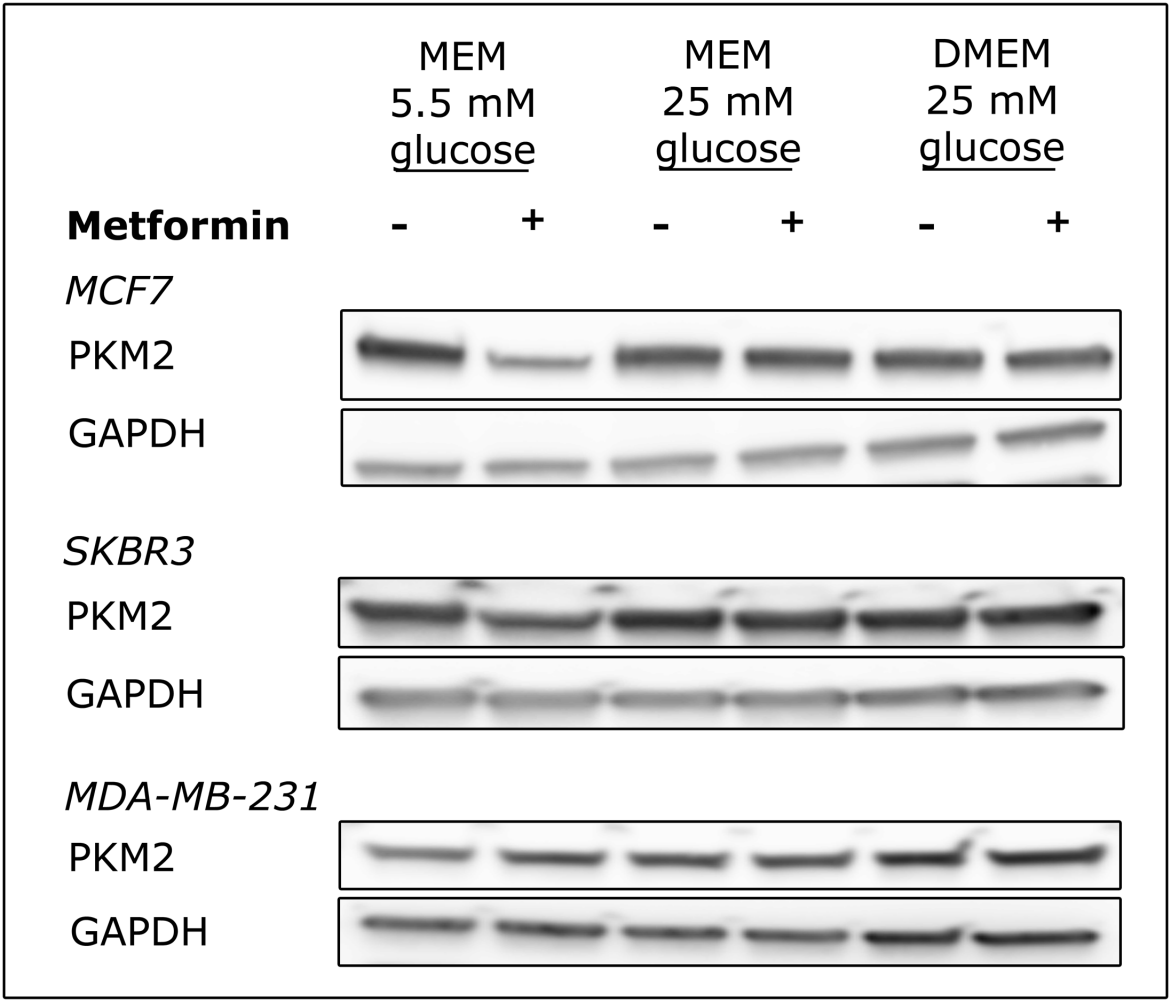
Metformin treatment induces a glucose-sensitive PKM2 downregulation in MCF7 and SKBR3 cells. MCF7, SKBR3 and MDA-MB-231 cells were plated at 8X10^5^ cells/well in 6-well plates in MEM medium with 5.5 mM or 25 mM glucose or DMEM and were treated with 10 mM metformin. After 48, 36 and 48 hours, respectively cells were lysed and protein extracts were analysed by Western Blot with an anti-PKM2 antibody. GAPDH was used as loading control.

Since we observed that metformin cytotoxicity was dependent on nutrient availability, we analysed if modulation of the glycolytic pathway was involved in this effect. We focused on Pyruvate Kinase M2 (PKM2), a key player in the modulation of the glycolytic pathway. Cells from the three models of breast cancer were treated with 10 mM metformin in the three different conditions previously described and the level of PKM2 was monitored by Western Blot. As shown in Fig 5, metformin induced a downregulation of PKM2 expression in both MCF7 and SKBR3 cells. This effect was completely abolished when cells were grown in media with non-limiting concentrations of glucose. No effect was observed in the resistant cell line MDA-MB-231 after 48 hours treatment (Fig 5). To investigate PKM2 downregulation at transcriptional level, gene expression was analysed by real-time PCR. Surprisingly, in MCF7 cells metformin treatment reduced PKM2 RNA expression levels in all the analysed conditions. This result indicates that metformin can control not only PKM2 transcription but also some post-translational regulatory mechanisms and that this latter effect is dependent on nutrient availability (S6 Fig, upper panel). The ability of metformin to induce protein downregulation at post-translational levels has been observed also in SKBR3 cells where a slight decrease in PKM2 protein expression was present (Fig 5) while RNA level was not modified after treatment (S6 Fig, middle panel). After 24 hours treatment no alteration in PKM2 protein expression was observed (data not shown) but a slight increase in mRNA is present. Finally, MDA-MD-231 cells showed no differences in PKM2 mRNA levels after 24 hours treatment while an initial upregulation of PKM2 was observed after prolonged incubation with the drug even if this was not statistically significant due to the high level of variability between biological replicates (S6 Fig, lower panel).

### Inhibition of mTOR by rapamycin is not sufficient to induce apoptosis and does not affect PKM2 levels

**Fig 6:**
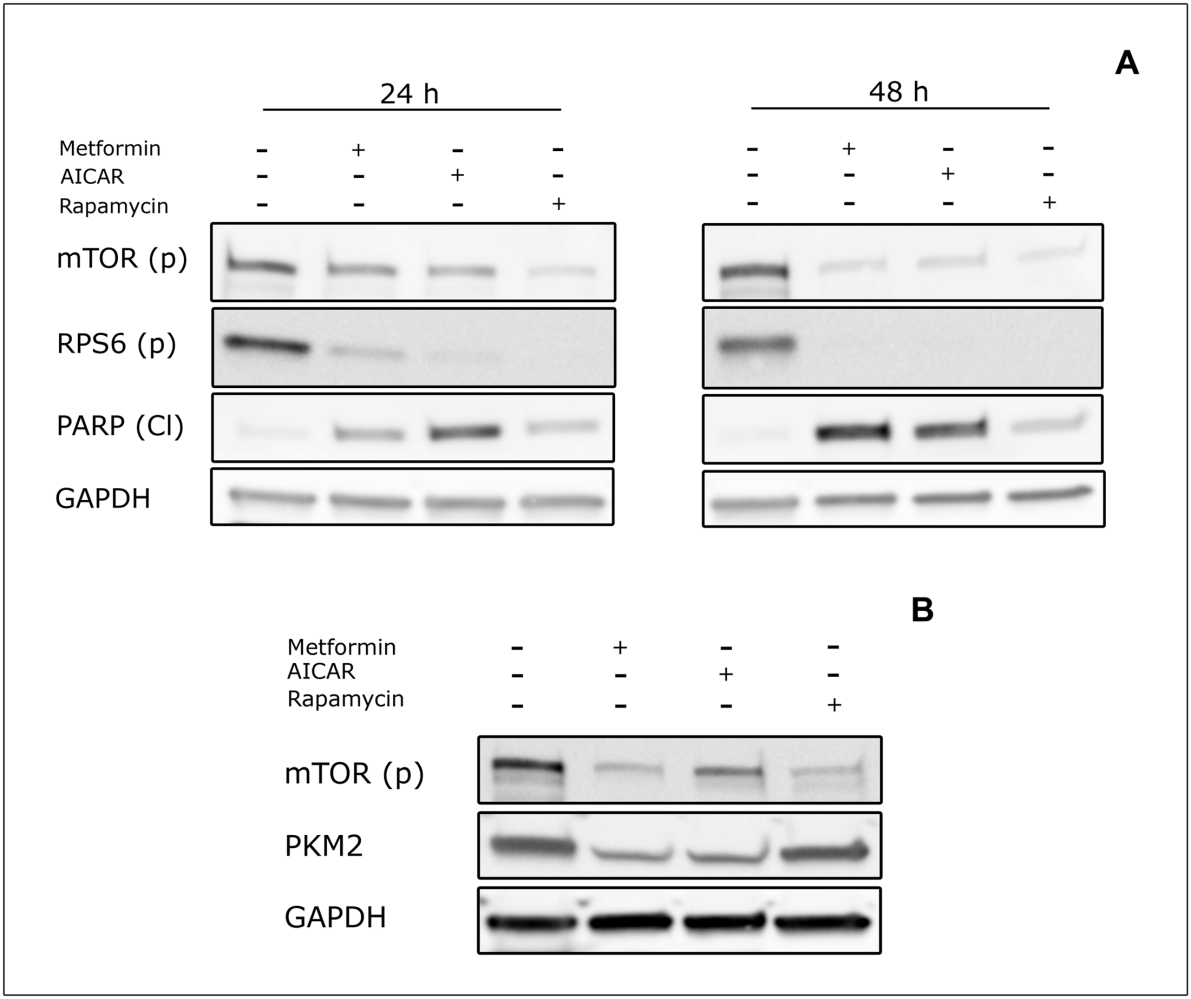
AMPK activation by AICAR is sufficient to induce apoptosis and downregulation of PKM2. A) MCF7 cells were plated at 8X10^5^ cells/well in 6-well plates in MEM medium and treated with 10 mM metformin, 3 mM AICAR, 100 nM rapamycin or PBS as control, for 48 hours. After treatment cells were lysed and protein extracts were analysed by Western Blot with antibodies directed against mTOR (p) Ser2448, RPS6 (p) Ser240/244 and PARP (Cl). GAPDH was used as loading control. B) MCF7 cells were plated at 8X10^5^ cells/well in 6-well plates in MEM medium and treated with 10 mM metformin, 3 mM AICAR, 100 nM Rapamycin or PBS as control for 48 hours. After treatment cells were lysed and protein extracts were analysed by Western Blot with anti-mTOR (p) Ser2448 and anti-PKM2 antibodies. GAPDH was used as loading control.

The most invoked mechanism underlying the cell-autonomous effect of metformin on cancer cells implies the ability to inhibit the mTOR pathway via the activation of AMPK. We asked whether this mechanism is involved not only in the metformin-induced reduction of cell proliferation but also in its pro-apoptotic effect. We first asked whether the direct activation of AMPK could induce apoptosis. Cells were plated in MEM medium at normoglycemic concentration and treated for 24 and 48 hours with 3 mM AICAR, an AMP analog that binds to and activates AMPK. As shown in Fig 6A, treatment with AICAR induced downregulation of mTOR phosphorylation at a level comparable to that achieved with metformin. After 48 hours treatment, PARP cleavage was higher than the control in both AICAR and metformin treated cells indicating that the induction of AMPK is sufficient to induce apoptosis in our experimental conditions (Fig 6A). Next we asked whether this effect could be recapitulated by mTOR inhibition. To this end cells were treated with 100 nM rapamycin for 24 and 48 hours. Although the treatment induced a significant downregulation of the mTOR pathway, as measured by phosphorylation of Ser2448 in mTOR and phosphorylation of the downstream target RPS6, it did not cause apoptosis (Fig 6A). All together these results indicate that sustained induction of AMPK is sufficient to induce apoptosis and that signalling routes, other than the ones inhibited by rapamycin, need to be perturbed in order to cause cell death.

Similarly, to determine if the AMPK-mTOR pathway was involved in metformin-induced PKM2 downregulation, the level of expression of the Pyruvate Kinase was analysed in cells treated with 10mM metformin, 3 mM AICAR or 100 nM rapamycin for 48 hours. Treatment with both metformin and AICAR induced a strong PKM2 downregulation while in rapamycin-treated cells its expression was not altered (Fig 6B). These data demonstrate that signalling pathways other the ones inactivated by rapamycin are necessary to downregulate PKM2.

## Discussion

Recent epidemiological, preclinical and clinical evidence in patients affected by diabetes and in healthy subjects called attention on a possible role for metformin as an anti-cancer agent [6]. Different studies have shown a correlation between a metformin regimen and a reduced cancer incidence [7,8]. Moreover, diabetic patients treated with metformin develop cancers characterized by a less aggressive phenotype [21,22]. These observations have led to several clinical trials aimed at determining the efficacy of metformin as an anticancer agent for different cancer types [23,24]. Several observations in isolated cell culture suggest diverse cell autonomous mechanisms that may favour anti-neoplastic effects of metformin, such as the modulation of PKCa/ERK and JNK/AP-1 signalling pathways [25]. The drug ability to inhibit cancer growth has been often correlated to the inhibition of the proliferative processes controlled by the mTOR pathway [26]. In addition to its anti-proliferative properties, some recent reports have shown that in *in vitro* cell cultures metformin has also an apoptotic effect [27,28]. However, the reported results are often contrasting and the mechanisms underlying this anticancer effect have not been clarified [14,15,17,29]. We set out to clarify the experimental conditions that modulate the anti proliferative and apoptotic effect of metformin *in vitro*. Here we have studied the response of breast cancer cell lines to metformin treatment in different experimental conditions.

First we observed that metformin caused cell death only in cells plated at high density, the only condition in which the cleaved forms of both Caspase 7 and PARP were detectable.

Since in most of the published reports the effect of metformin on cancer cells is observed after 24 hours treatment [14,15,27], we asked whether the treatment time could influence metformin cytotoxicity. By prolonging the treatment to 48 hours the number of dead cells increases up to 60%. The observation that nutrient replenishment by addition of fresh medium after 24 hours treatment can limit metformin cytotoxicity suggest that nutrient availability plays a major role in the modulation of the apoptotic effect.

We first confirmed that metformin is cytotoxic in growth conditions where glucose is limiting [18,19,29]. Interestingly we observed that, by increasing glucose availability, it was possible to limit metformin cytotoxicity without significantly modulating the downregulation of mTOR. To ascertain whether additional nutrients, other than glucose, influence cell sensitivity to metformin, we compared the effect of the treatment in different growth media. After 48 hours in 10 mM metformin, 80% of cells grown in MEM, a nutrient-poor medium, were dead as shown by staining with Trypan Blue. Conversely by culturing in DMEM medium, a commonly used growth medium containing 25 mM glucose and a richer supply of amino acids, the number of dead cell was reduced to less then 10%. The observation that metformin cytotoxicity was lower in DMEM than in MEM at comparable glucose concentrations, suggested that additional nutrients, other than glucose, affect metformin cytotoxicity. Differently from what was observed by increasing the concentration of glucose, only the culturing in DMEM medium reduced the inhibitory effect of metformin on the mTOR pathway. These data are consistent with a model whereby the metformin pro-apoptotic effect is influenced by glucose availability while mTOR pathway inhibition is mediated by alternative mechanisms that are mainly influenced by other nutrients required for cell growth. We attempted to identify these “nutrients”, by adding to the MEM medium amino acids known to be important for energy production and mTOR activity. However, we were not able to rescue cells from metformin induced apoptosis by supplementing the medium with glutamine, serine and leucine (data not shown), indicating that the shortage of these amino acids, as single components, are not responsible for metformin cytotoxicity.

We hypothesize that metformin causes an alteration of the cellular energy homeostasis thereby inducing a rewiring of metabolic pathways that drives cells to use alternative systems to produce energy. When cells are grown in high-nutrient media this rewiring allows the cell to survive while when nutrients are not supplied in excess, cells are not able to produce sufficient amounts of energy and undergo apoptosis. Further analyses are needed to better understand which are the main metabolic pathways responsible for this effect.

We next investigated the generality of these phenomena by testing the sensitivity to metformin treatment of two additional models of breast cancer cells, SKBR3, which overexpresses the HER2/c-erb-2 gene product, and MDA-MB-231, which are triple negative. Both cell lines, similarly to MCF7, underwent apoptosis after metformin treatment in nutrient-poor medium while they became more resistant when grown in a nutrient-rich medium. The two cell lines, however, displayed a different sensitivity to the treatment: 36 hours were sufficient to induce robust cell apoptosis and PARP cleavage in SKBR3, while MDA-MB-231 even after 48 hours treatment showed limited signs of apoptosis.

All together these data indicated that nutrient availability influence metformin cytotoxicity not only in MCF7 cells but also in other breast cancer cell models and, more important, in the more aggressive MDA-MB-231. Moreover, these results suggested that for some cellular models prolonged treatments are needed to induce cell death. Other groups previously analysed the effect of metformin treatment on these cellular models but the results are difficult to compare and often contradictory [14,15,17,29]. Our work stresses the importance of defining standard conditions and underlines nutrient availability as an important factor modulating the anti-cancer effect of metformin.

The effect of metformin on MDA-MDA-231 was previously analysed by Deng et al who demonstrated that 48 hours metformin treatment of triple negative breast cancer cells induced apoptosis and that this effect was mediated by inactivation of Stat3 [30]. Interestingly, it was recently shown that PKM2 can act as a protein kinase in the nucleus phosphorylating Stat3 and promoting its activity [31]. Considering these data together with our results, we can hypothesize that metformin cytotoxicity is mediated by a downregulation of PKM2 expression that in turn translates in an inhibition of Stat3 activity and induction of cellular apoptosis. This hypothesis needs further experiments to be confirmed. A similar scenario is depicted by Blandino et al., who have characterized miRNAs upregulated or downregulated in breast cancer cells by metformin. Two miRNAs, characterized by others to silence PKM2, were found upregulated, while three miRNAs targeting SOCs RNAs are downregulated [32]. This context suggests that metformin induces inhibition of Stat3 by multiple posttranslational mechanisms.

Considering the effect of glucose supply on metformin cytotoxicity, we contemplated the possibility that the biguanide apoptotic effect could be modulated by the activity of the glycolytic pathway. To this end we monitored the expression of the isoform M2 of Pyruvate Kinase (PKM2), a key enzyme in the glycolytic pathway. The splicing isoform M2 of the PKM gene is mainly expressed in cells characterized by a high rate of nucleic acid synthesis, such as those in developing tissues and in tumors [33–35]. We observed that after 48 hours metformin was able to reduce PKM2 transcription in MCF7 cells, but interestingly this effect produced a downregulation of protein expression only when cells were grown in nutrient poor medium. This effect was observed also in SKBR3 cells where no reduction in mRNA expression was present but a slight decrease in protein expression after 36 hours treatment was induced only when cells where grown in nutrient poor medium. Taken together, these data suggest that metformin can control PKM2 transcription and that, more important, it activates some post-translational regulatory mechanisms that are dependent on nutrient availability. Our data indicate that metformin affects PKM2 expression by controlling both transcription and post-transcriptional mechanisms and that this effect is cell type dependent.

The multi functional capacities of PKM2 and its role in tumors are intricate and not well understood [36,37]. This makes it difficult to predict the consequences of downregulating this enzyme isoform, as we observed after metformin treatment. Our results revealed a correlation between downregulation of this enzyme isoform and induction of apoptosis. This is in agreement with the observation that siRNA knockdown of PKM2 caused increased apoptosis in multiple cancer cell lines [38].

Our data are consistent with reports that correlate the metformin anti-tumor effect to an alteration of cellular energy metabolism. In particular it has been shown that the combined use of 2-deoxyglucose (2DG), a glycolysis inhibitor, and metformin treatment have a synergistic effect on cytotoxicity [39]. Moreover, Marini et al [40] showed that metformin reduces hexokinase activity in MDA-MB-231 cells causing an alteration in the energetic homeostasis that leads to cell death. Recently, Janzer A and colleagues studied the metabolomic profile of a Src-inducible model of breast cancer transformation after biguanides treatment. They reported that metformin decreases the level of tricarboxylic acid (TCA) cycle metabolites and they proposed an increase in the conversion of pyruvate, which directly feeds the TCA cycle, to lactate as a possible cause of the observed decrement in pyruvate concentration [41]. Our data are consistent with an alternative mechanism whereby the reduction of pyruvate levels maybe due to a decrease of PKM2 level rather than an increase in pyruvate conversion to lactate.

While this manuscript was under revision, Chen G and colleagues published an interesting manuscript where they confirmed, in a different cancer model, our results about the metformin ability to downregulate PKM2 expression. They showed that metformin was able to induce apoptosis in gastric cancer cells and that this effect was mediated by HIFalpha/PKM2 downregulation. These data, together with ours, suggest that metformin ability to modulate PKM2 expression is not limited to breast cancer but represents a general mechanism of metformin cytotoxicity [42].

**Fig 7:**
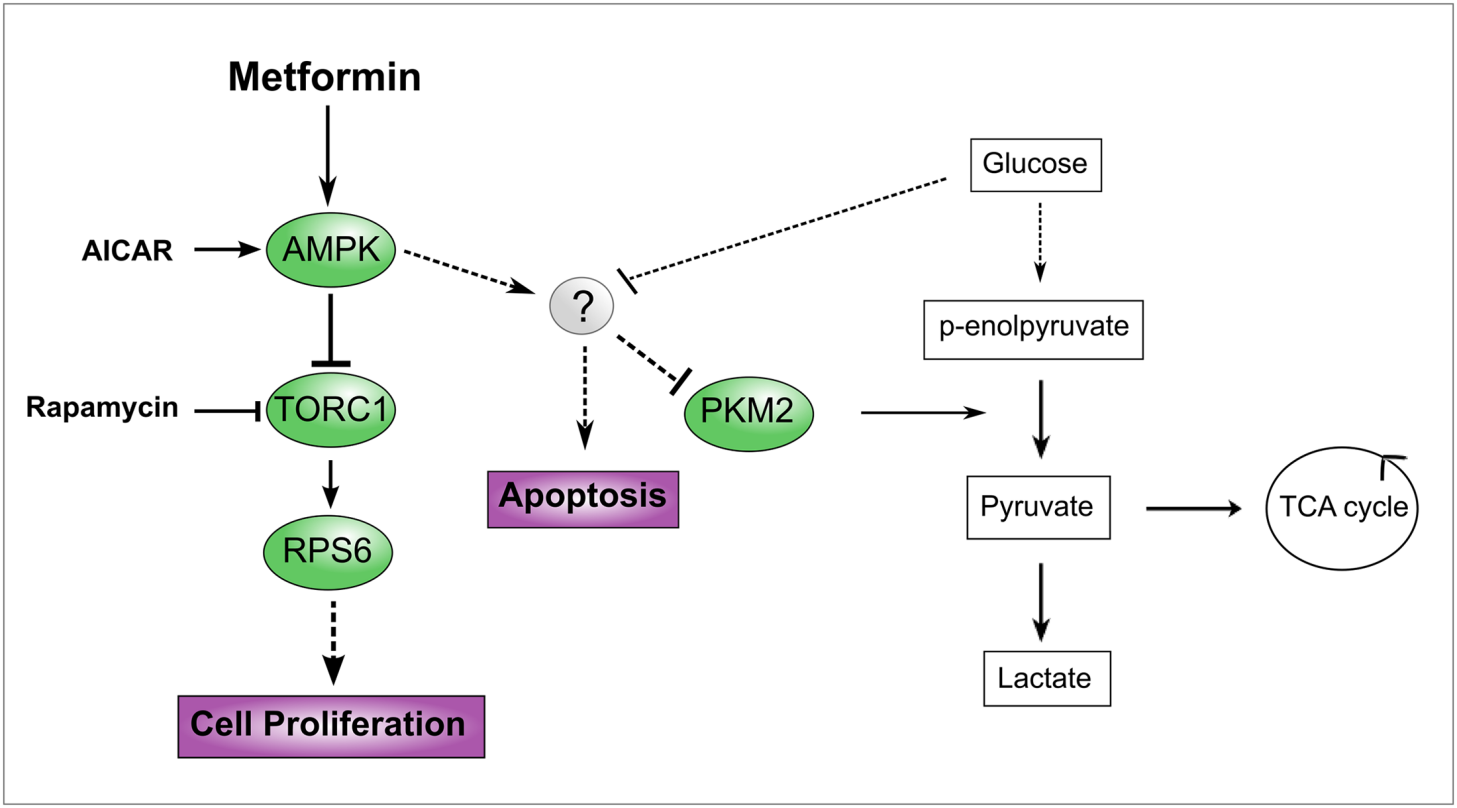
Model consistent with the data presented in this manuscript. The model represents the logic relationships that are supported by the experimental observations presented here. Solid and broken lines in the left “signalling” part of the model represent direct and indirect interactions, respectively. Activations and inhibitions are depicted as arrowhead and hammerhead. On the right “metabolic” part of the model arrows represents metabolite conversions. The circle with a question mark represents the unknown entity that mediate the downregulation of PKM2 when AMPK is activated at high glucose concentration.

The most studied effect of metformin is the inhibition of cancer cell proliferation mediated by the inactivation of the mTOR pathway following AMPK activation [43]. Using AICAR, a direct activator of AMPK, and rapamycin, an inhibitor of the TORC1 complex, we investigated the involvement of these two key regulators of growth pathways in metformin-induced apoptosis. In our experimental model the direct activation of AMPK led to cellular apoptosis up to levels that were comparable to those achieved by metformin treatment demonstrating that the activation of AMPK is by itself sufficient to induce cell death when nutrients are limiting. Rapamycin on the other hand, was not effective in inducing apoptosis suggesting that TORC1 inhibition by rapamycin is not sufficient to activate programmed cell death. Together, these data suggest that metformin-induced apoptosis is mediated by AMPK activation via routes that do not involve modulation of mTOR activity by its allosteric inhibitor rapamycin (see model in Fig 7). Moreover, we demonstrated that PKM2 downregulation is also mediated by AMPK activation while it is independent of mTOR inhibition. The characterization of the molecular mechanisms that link AMPK activation and PKM2 downregulation and the demonstration that PKM2 downregulation is required in order for metformin to induce apoptosis need further experiments. Our data are in agreement with recent findings by Zhuang and colleagues [29] that reported that metformin induces reduction of ATP production only in low glucose medium, thus suggesting that metformin cytotoxicity is strictly dependent on nutrient shortage.

## Conclusions

Our results emphasize a correlation between metformin cytotoxicity and nutrient availability. These data call attention on the importance of carefully controlling the experimental conditions while studying the mechanism of action of metformin and other drugs that affect cellular metabolism. Moreover our work highlight for the first time that metformin treatment modulates the glycolytic pathway by downregulating PKM2 levels, through an AMPK dependent mechanism. These data suggests that modulation of PKM2 expression or activity to reduce upstream glycolytic intermediates could be a promising strategy to boost the metformin anti-cancer effect. Further experiments are needed to understand the mechanism through which metformin induces PKM2 downregulation and whether additional metabolic pathways, other then glycolysis, are downregulated by the treatment thus inducing a lack of cellular energy production.

## Supporting Information

S1 Fig Total protein expression after metformin treatment in MCF7 cells plated at different cell densities. MCF7 cells were plated in MEM medium at different densities from 2X10^5^ to 16X10^5^ cell/well in 6-well plates. Cells were then treated with 10mM metformin or with PBS as control for 24 hours. After treatment cells were lysed and protein extracts were analysed by Western Blot with antibodies directed against mTOR, RPS6, Caspase 7 and PARP. GAPDH was used as loading control.
(TIF)

S2 Fig Total protein expression after metformin treatment in MCF7 cells grown in MEM medium with increasing amounts of glucose. MCF7 cells were plated at 8X10^5^ cells/well in 6-well plates in MEM medium with increasing amounts of glucose from 5.5 mM to 25 mM and treated with 10 mM metformin for 48 hours. After treatment cells were lysed and protein extracts were analysed by Western Blot with antibodies directed against mTOR, RPS6 and PARP. GAPDH was used as loading control.
(TIF)

S3 Fig Total protein expression after metformin treatment in MCF7 cells grown in MEM or DMEM media. MCF7 cells were plated at 8X10^5^ cells/well in 6-well plates in MEM medium with 5.5 mM or 25 mM glucose or DMEM and treated with 10 mM metformin for 48 hours. After treatment cells were lysed and protein extracts were analysed by Western Blot with antibodies directed against mTOR, RPS6 and PARP. GAPDH was used as loading control.
(TIF)

S4 Fig Analysis of metformin induced apoptosis by Annexin V/PI double staining. A) Dot plot of flow cytometric analysis of apoptotic cells after 24 (upper panel) and 48 hours (lower panel) treatment. Cell populations: alive cells (annexin V negative, PI negative), early apoptotic cells (annexin V positive, PI negative), late apoptotic cells (annexin V positive, PI positive), necrotic cells (annexin V negative, PI positive). B) Bar graph quantifying the percentage of early and late apoptotic cells after 24 (right panel) and 48 hours (left panel) treatment. Data reported is the mean of two independent experiments.
(TIF)

S5 Fig Total protein expression after metformin treatment in SKBR3 and MDA_MB-231. SKBR3 and MDA-MB-231 cells were plated at 8X10^5^ cells/well in 6-well plates in different growth media (MEM 5.5 mM glucose, 25 mM glucose and DMEM) in 6-well plates treated with 10 mM metformin for 24h, and 36h or 48 hours, respectively. After treatment cells were lysed and protein extracts were analysed by Western Blot with antibodies directed against mTOR, RPS6 and PARP. GAPDH was used as loading control.
(TIF)

S6 Fig PKM2 mRNA expression after treatment with metformin. After 24 and 48 or 36 hours cells were lysed and PKM2 mRNA expression was analysed by real-time PCR. RNA levels were reported as fold change of metformin treated samples to the control PBS treated samples. Beta-actin was used as endogenous control for sample normalization. Data reported is the mean of three independent experiments.
(TIF)
