# Supplementary figures and images for: Metformin Induces Apoptosis and Downregulates Pyruvate Kinase M2 in Breast Cancer Cells Only When Grown in Nutrient-Poor Conditions
Source: PLoS One. 2015 Aug 20;10(8):e0136250. doi: 10.1371/journal.pone.0136250 (PMC4546379; doi:10.1371/journal.pone.0136250)

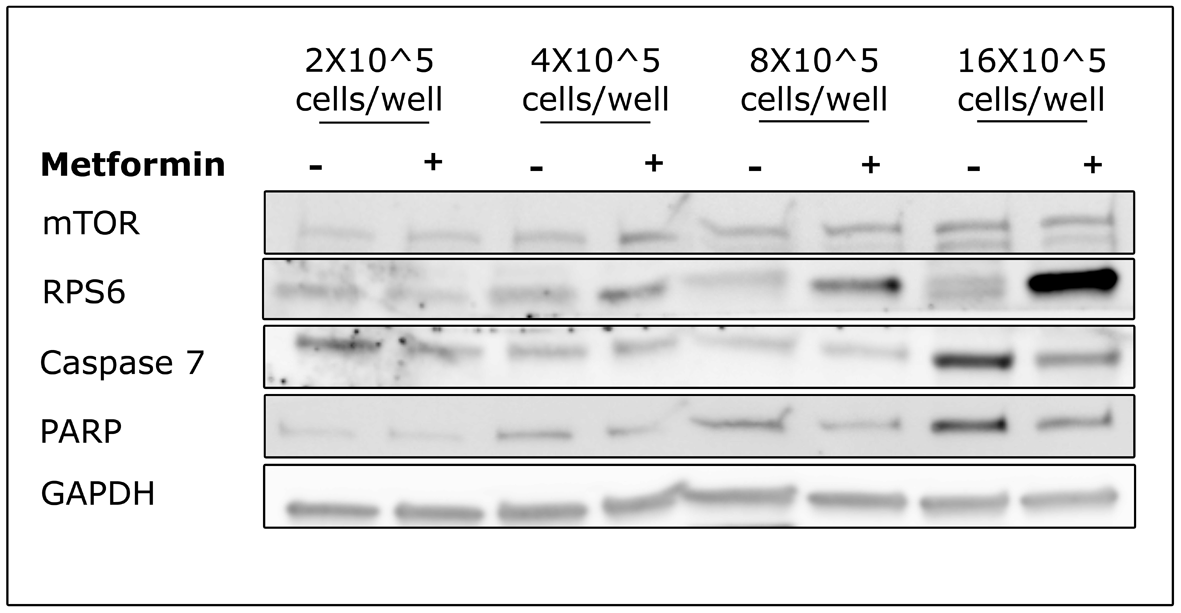

Supplement: S1 Fig — MCF7 cells were plated in MEM medium at different densities from 2X105 to 16X105 cell/well in 6-well plates. Cells were then treated with 10mM metformin or with PBS as control for 24 hours. After treatment cells were lysed and protein extracts were analysed by Western Blot with antibodies directed against mTOR, RPS6, Caspase 7 and PARP. GAPDH was used as loading control. (TIF) [file pone.0136250.s001.tif]

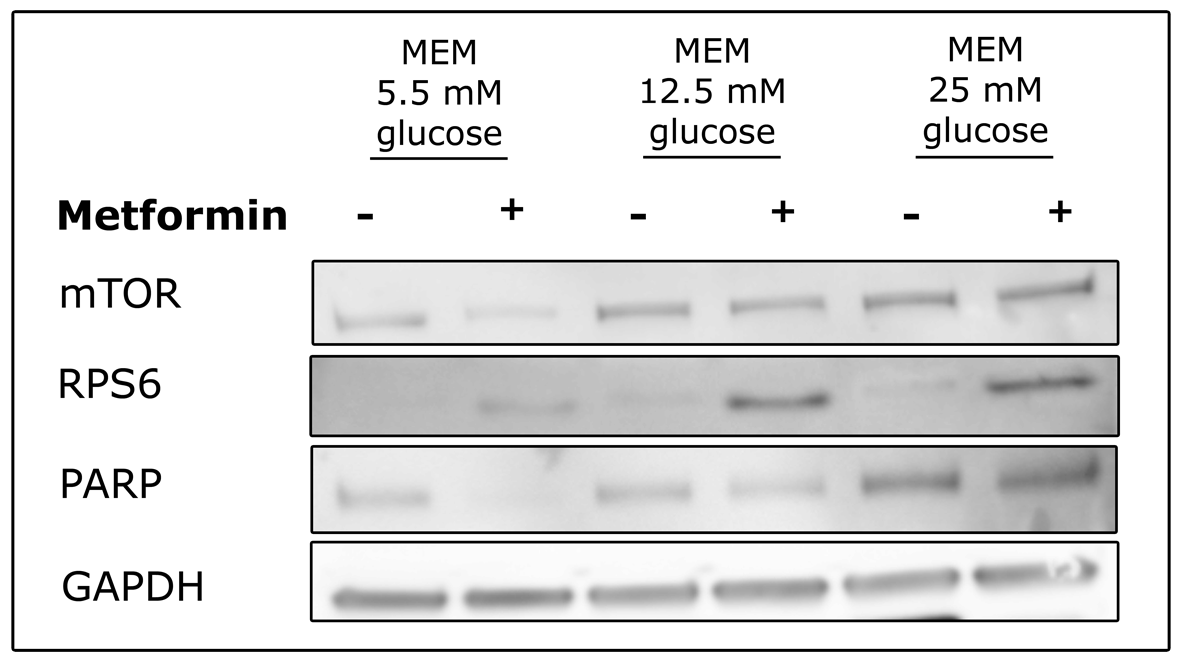

Supplement: S2 Fig — MCF7 cells were plated at 8X105 cells/well in 6-well plates in MEM medium with increasing amounts of glucose from 5.5 mM to 25 mM and treated with 10 mM metformin for 48 hours. After treatment cells were lysed and protein extracts were analysed by Western Blot with antibodies directed against mTOR, RPS6 and PARP. GAPDH was used as loading control. (TIF) [file pone.0136250.s002.tif]

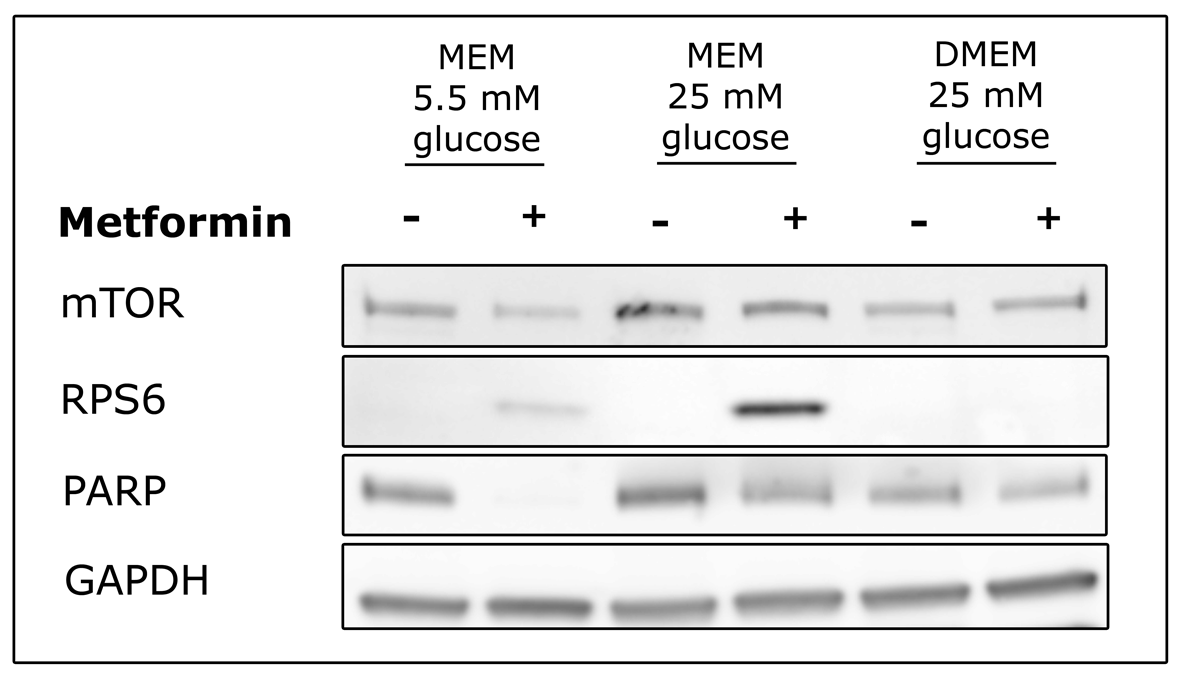

Supplement: S3 Fig — MCF7 cells were plated at 8X105 cells/well in 6-well plates in MEM medium with 5.5 mM or 25 mM glucose or DMEM and treated with 10 mM metformin for 48 hours. After treatment cells were lysed and protein extracts were analysed by Western Blot with antibodies directed against mTOR, RPS6 and PARP. GAPDH was used as loading control. (TIF) [file pone.0136250.s003.tif]

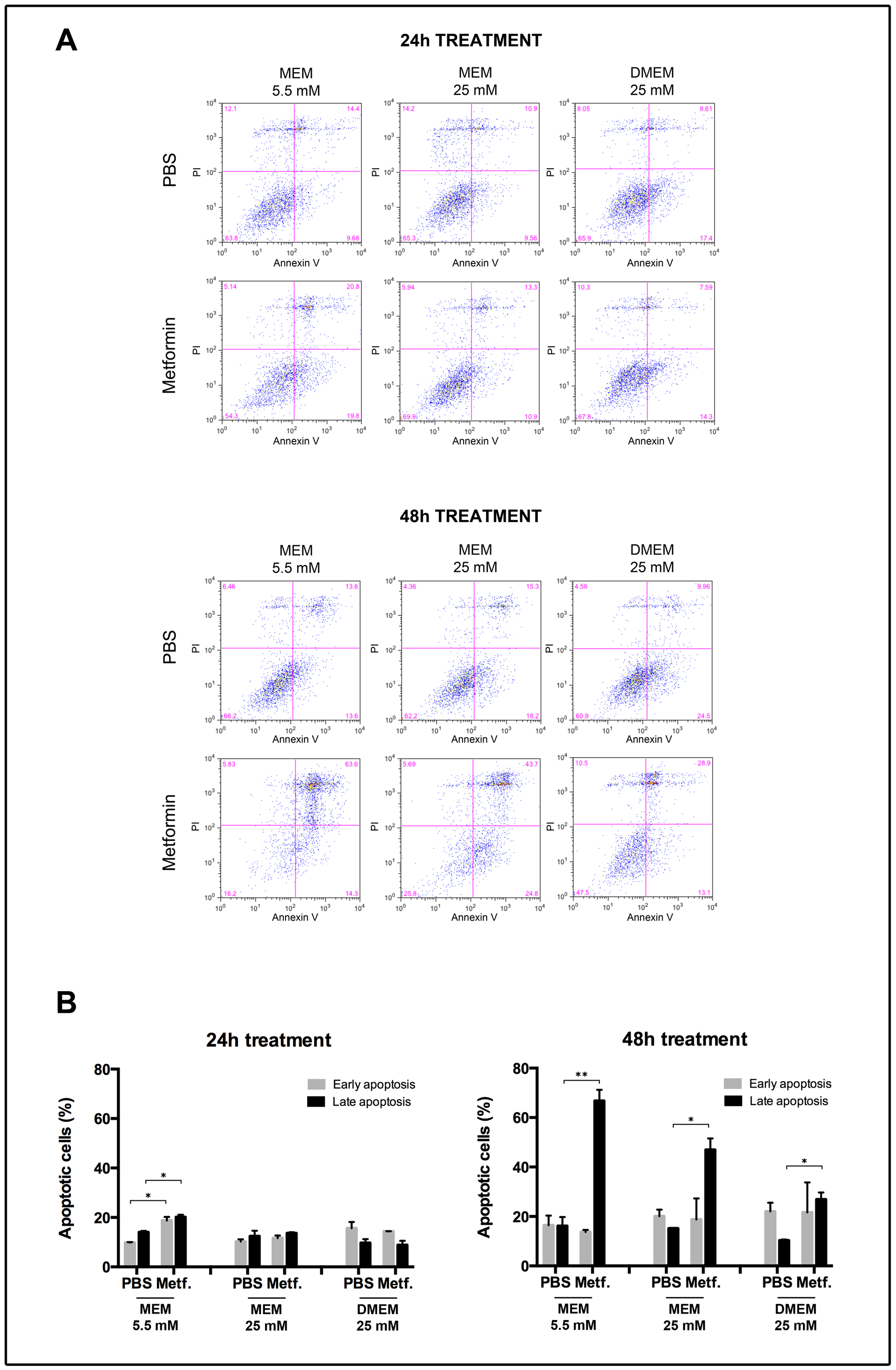

Supplement: S4 Fig — A) Dot plot of flow cytometric analysis of apoptotic cells after 24 (upper panel) and 48 hours (lower panel) treatment. Cell populations: alive cells (annexin V negative, PI negative), early apoptotic cells (annexin V positive, PI negative), late apoptotic cells (annexin V positive, PI positive), necrotic cells (annexin V negative, PI positive). B) Bar graph quantifying the percentage of early and late apoptotic cells after 24 (right panel) and 48 hours (left panel) treatment. Data reported is the mean of two independent experiments. (TIF) [file pone.0136250.s004.tif]

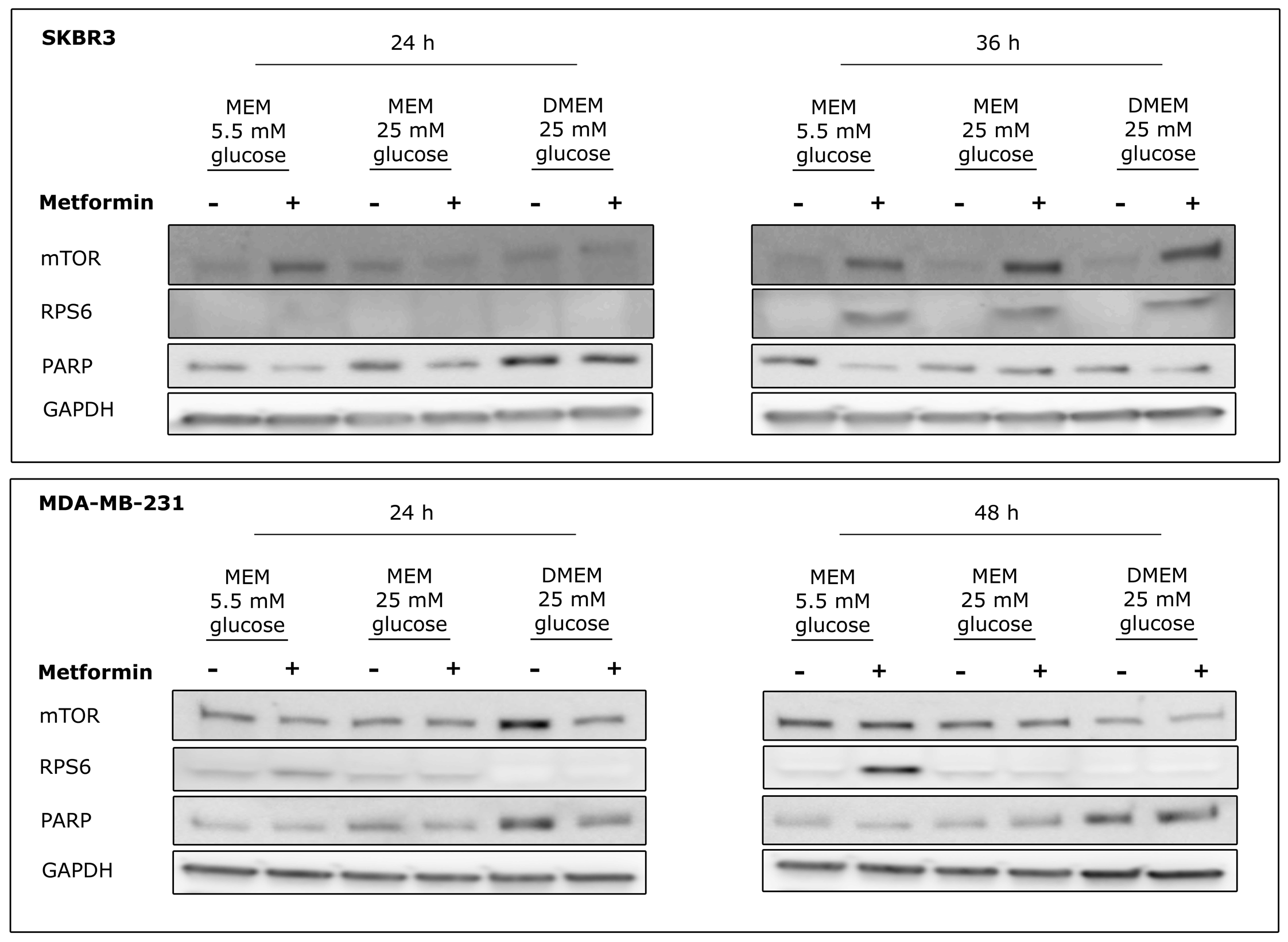

Supplement: S5 Fig — SKBR3 and MDA-MB-231 cells were plated at 8X105 cells/well in 6-well plates in different growth media (MEM 5.5 mM glucose, 25 mM glucose and DMEM) in 6-well plates treated with 10 mM metformin for 24h, and 36h or 48 hours, respectively. After treatment cells were lysed and protein extracts were analysed by Western Blot with antibodies directed against mTOR, RPS6 and PARP. GAPDH was used as loading control. (TIF) [file pone.0136250.s005.tif]

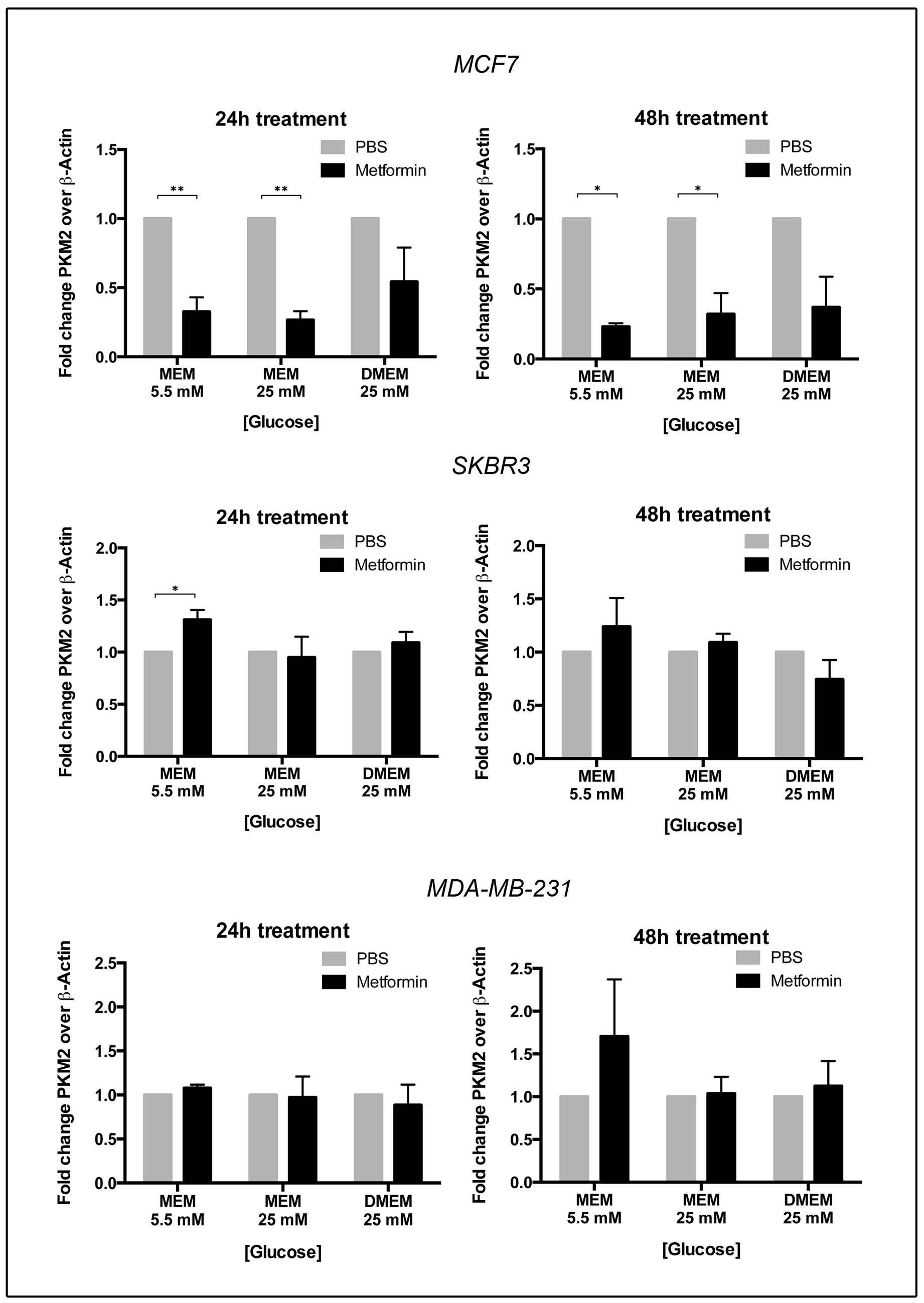

Supplement: S6 Fig — After 24 and 48 or 36 hours cells were lysed and PKM2 mRNA expression was analysed by real-time PCR. RNA levels were reported as fold change of metformin treated samples to the control PBS treated samples. Beta-actin was used as endogenous control for sample normalization. Data reported is the mean of three independent experiments. (TIF) [file pone.0136250.s006.tif]
